# Biosynthesis of bacterial cellulose nanofibrils in black tea media by a symbiotic culture of bacteria and yeast isolated from commercial kombucha beverage

**DOI:** 10.1007/s11274-022-03485-0

**Published:** 2022-12-20

**Authors:** Doaa A. Hamed, Heba H. Maghrawy, Hussein Abdel Kareem

**Affiliations:** National Center for Radiation Research and Technology (NCRRT), Radiation Microbiology Department, Egyptian Atomic Energy Authority (EAEA), Cairo, Egypt

**Keywords:** SCOBY, Ethanol, *Acinetobacter*, Candida, Pichia, Gamma irradiation

## Abstract

Bacterial cellulose has drawn the attention for its unique properties and applications including; medicine, pharmacy, food, agricultural, textile and electronics. The present study focused on the production of bacterial cellulose nanofibrils (BCNF) from black tea as cost effective alternative medium in addition to study the effect of gamma radiation on BCNF properties. A symbiotic culture of bacteria and yeast (SCOBY) were isolated from commercial Kombucha beverage and were identified as *Acinetobacter* *lowffii* and *Candida krusei*, respectively. The symbiotic culture was used for production of BCNF on Hestrin-Schramm (HS), black tea (BT) and modified BT media. BCNF was purified (0.5 N NaOH) and quantified by dry weight, yield and productivity determination. Characterization and effect of gamma radiation (5–25 kGy) on BCNF were studied using Scanning Electron Microscope (SEM), Fourier transform infrared (FTIR) and X-Ray Diffraction (XRD). The highest BCNF production was achieved using BT medium with 0.2% tea and 6.0% commercial sugar (with dry weight 4.77–4.61 g/l and productivity 68.14% and 65.85%, respectively). Supplementation of BT medium with 1% ethanol, 0.27% Na_2_HPO_4_ and 0.5% yeast extract individually, enhanced the BCNF production (7.85, 6.84 and 5.73 g/l), respectively. FTIR spectrum of BCNF from sugared water (SW), HS and BT showed similar structure with high purity. As a conclusion, gamma irradiation has no effect on the BCNF structure while showed different effects on its crystallinity index and size with the different doses. The changes in CrI were ranged between (17 and 23.5%), while the crystallinity size (Cs) was affected by gamma irradiation in a positive relationship where the crystalline size was decreased (33%) by exposure to 5 kGy then increased by increasing the dose of radiation reaching 25.7% at 25 kGy. SEM graphs showed the morphology of microbial culture and its symbiotic relationship in addition to the ultrafine structure of non-irradiated and irradiated BCNF.

**Graphical abstract:**

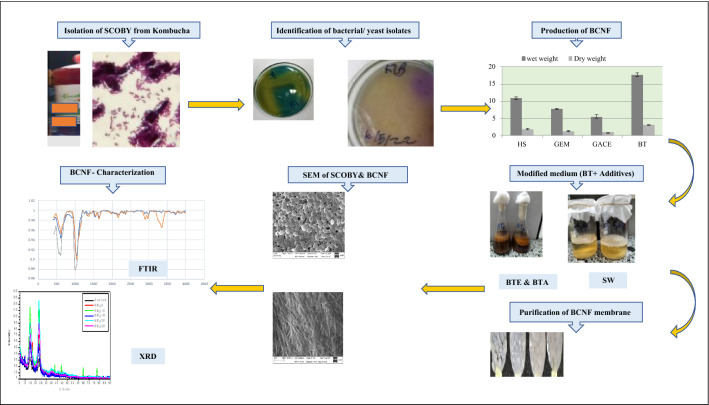

## Introduction

Cellulose is one of the most abundant biomaterials on the earth and representing the essential component of plant cell wall. It is produced by some bacterial species as secondary metabolite, water-insoluble, exopolysaccharide with the same chemical formula of plant cellulose (C_6_H_10_O_5_)_n_. It is formed as a floating solid polymeric film at the air–liquid interface of the culture medium during fermentation process of kombucha tea (Villarreal-Soto et al. 2019).

Kombucha is a traditional beverage made from sugared tea, *Camellia sinensis* plant, fermented by symbiotic cultures of bacteria and yeast (SCOBY) that consist of a complex microbial consortium including; acetic acid, lactic acid bacteria and yeast (Arıkan et al. 2020). Different genera of bacteria were m4entioned as cellulose producers, such as: *Acetobacter, Gluconacetobacter, Komagataeibacter*, *Gluconobacter, Rhizobium, Agrobacterium, Aerobacter, Achromobacter, Azotobacter, Salmonella, Escherichia, Sarcina* and *Pseudomonas*. While, a wide diversity of yeasts such as *Saccharomyces*, *Zygosaccharomyces*, *Schizosaccharomyces*, *Dekkera*/*Brettanomyces*, *Pichia* and *Candida* were included in Kombucha SCOBY (Jayabalan et al. 2014; Bielecki et al. 2002; Laureys et al.^2020^).

Generally, the formation of BC pellicle/membrane in the air–liquid interface of the media was explained by the need of aerobic bacteria to reach to oxygen in the surface, to tolerate the stress conditions resulted from high acid and ethanol concentrations, to create a protection barrier against UV radiation and to allow moisture retention to prevent dehydration. BC is considered as a promising biomaterial possesses unique properties compared with plant cellulose; like: (a): its high chemical purity due to its highly pure fiber structure free from hemicellulose, pectin and lignin (Mohite and Patil 2014), (b): its nano-fibrils structure from 3 to 8 nm (Park et al. 2009), (c): the simplicity of its isolation and purification (Keshk 2014). Moreover, it has high-water absorption and retention capacities owing to its hydrophilic nature in addition to its high crystallinity, high mechanical strength, biodegradability and biocompatibility. All of these characteristics widely support the application of BC in several fields including; medicine, pharmacy, food, agricultural, textile and electronics.

The most restricted factor in BC commercial production is the high cost of fermentation media accompanied with low production yield. In order to overcome this problem, the attention was directed towards using economically alternative media from cheap sources in order to make the process less expensive and sustainable as well. Therefore, the present study focused on the production of bacterial cellulose nanofibrils (BCNF) in black tea (BT) as a cost-effective medium by a symbiotic culture of bacteria and yeast isolated from commercial Kombucha beverage. Moreover, the effect of different doses (5–25 kGy) of gamma radiation, as sterilization technique, on BCNF properties was investigated. The structure and crystallinity and morphological characteristics were determined using FTIR, XRD and SEM.

## Materials and methods

### Collection of samples

Kombucha beverage, packages of black tea powder (Lipton) and commercial white granulated sugar (sucrose) were purchased from local markets in Cairo, Egypt. Kombucha beverage were stored at 4 ℃ till further investigation.

### Culture media

#### Basal, selective, differential and enrichment culture media

Hestrin and Schramm (HS) was used as a basal medium for cultivation and production of BCNF. Several other media such as GYC, Herellea, Carr, GEM and GACE media were used as selective, differential, enrichment and production media as illustrated below in Table 1.

**Table 1:** Basal, selective, differential and enrichment culture media

Media	Type/use	Components (%)	References
Hestrin- Schramm (HS)	Basal	Glucose (2.0%)- yeast extract (0.5%)- Peptone (0.5%)- Na_2_HPO_4_ (0.27%)-Citric acid (0.115%)- Agar (2.0%)	Hestrin and Schramm (1954)
Glucose Yeast extract- Calcium Carbonate (GYC)	Selective	Glucose (2.0%)- yeast extract (1.0%)- CaCO_3_ (1.0%)- Agar (20.0%)	Swings (1992)
Herellea medium (HM)	Selective	Tryptone (1.5%)- Soya peptone (0.5%)- Sodium chloride (0.5%)- Lactose (1.0%)- Maltose (1.0%)- Bile salts mixture (0.125%)- Bromocresol purple (0.002%)- Agar (2%)	Karin and Kain (2006)
Carr medium	Differential	Yeast extract (3.0%)- Ethanol 95% (2.0%)- Bromocresol green (0.0022%)- Agar (2.0%)	Mandel et al. (1964)
Glucose-Ethanol medium (GEM)	Enrichment- Production	Glucose (1.5%)- Peptone (0.3%)- Yeast extract (0.3%)- Ethanol (0.5%)	Hanmougjai et al. (2007)
Glucose-acetic acid- citric acid- ethanol (GACE)	Cultivation/Maintenance	Glucose (1.0%)- Yeast extract (1.0%)- Peptone (0.7%)- Citric acid (0.02%)- Acetic acid (0.01%)- Ethanol (1.0%)	Revin et al. (2020)
Sabouraud Dextrose agar (SDA)	Yeast cultivation	Dextrose (4.0%)- Peptone (1.0%)- Agar (2.0%)	Bridson (2006)

#### Alternative and modified media for BCNF production

Sugared black tea was used as alternative medium for BCNF synthesis. Different concentrations of black tea (0–1%) and sugar concentration (0–10%) was tested for high BCNF production. The modified media were prepared by the addition of some components into BT media as mentioned below in 2.5 and Table 3. These additives represented the components of HS and GACE media (other than glucose) avoiding the repetition in components.

### BC producing strains

#### Primary screening of BC producing strain

1 ml of Kombucha beverage was inoculated under aseptic conditions in 100 ml HS basal medium in 250 ml conical flasks and incubated statically at 30 ℃ for 10–15 days for white film/pellicle formation on the surface of broth medium.

#### Isolation of bacteria and yeast from Kombucha beverage

A serial dilution (up to 10^–6^) from the flasks in the previous step was carried out for isolation of bacteria and yeast on HS agar medium using pour plate method. The bacterial isolate was sub-cultured on HS medium with antifungal agent, while yeast isolate was inoculated on Sabouraud Dextrose agar for further identification.

#### Selective media for identifying acid producing bacteria

The bacterial colonies were inoculated on plates of (GYC) medium, incubated at 30 °C and checked along 2–4 days. The colonies surrounded by clear zones were considered as confirmation step of acid producing strain.

#### Differential media

Carr medium was used in the presence of bromocresol green for distinguish between *Acetobacter* and *Gluconobacter*. While, HM was used for isolation and distinguishing of *Acinetobacter* species. The plates were incubated at 30 °C and checked during 2–4 days for the appearance of colonies and the changes in the color of medium.

#### Identification of BCNF producing strain

Cell morphology of bacterial and yeast isolates were stained and examined by light microscope and SEM. The strains were characterized using VITEK2 rapid biochemical test (Model: VITEC 2 COMPACT, BIOMÉRIEUX) in Al Mokhtabar Lab, Cairo, Egypt.

### Pre-inoculum preparation

The inoculum culture was prepared by transferring a single colony of the SCOBY growing on HS agar medium to a test tube containing 5 ml of HS broth medium and incubated at 30 °C for 48 h. The contents of the tube were transferred to 250 ml conical flask with 100 ml of HS and incubated at 30 °C for 48 h in shaking incubator (150 rpm). Each time the medium was inoculated with 5% (v/v) of 2 days pre-culture and incubated for 10 days at 30 °C.

### Production of BCNF in basal, alternative and modified media

The basal medium HS was compared with GEM and GACE media for maximum BC production. BT medium with different concentrations of tea (0–1.0%) and sugar (0–10%) were also examined. Modification on BT medium was carried out by addition of the components of HS and GACE media (other than glucose) at the same concentration and avoiding the repetition of the constituents as mentioned in details in table 3. All HS components other than glucose were added into BT medium (BTHS), while yeast extract (0.5%), Peptone (0.5% (, Na_2_HPO_4_ (0.27%), Citric acid (0.115%), Acetic acid (0.01%) and Ethanol (1.0%) were also added individually as indicated from BT1 to BT6, respectively. The culture conditions of all the tested media were adjusted to pH (5.5–6.0), 5% inoculum size and 100 ml working volume in 250 ml conical flasks at 30 °C for 10 days under static conditions.

### Production of BC using SCOBY without sterilization

BT media was prepared as mentioned before and divided in two clean conical flasks (100 ml/ 250 ml) washed with boiling tap water and ethyl alcohol (70%) (Jayabalan 2014). The flasks were inoculated under aseptic conditions by 5 ml from the original bottle of Kombucha beverage (1 ml of ethanol was added to one of the flasks), then incubated statically at 30 °C for 10 days and checked periodically.

### Purification and quantitative determination of BCNF

Gelatinous BCNF formed on the surface of broth media were harvested carefully and washed several times by distilled water, incubated in a water bath for 30 min in 0.5 N NaOH at 80 °C, then neutralization of the filtrate by 4% acetic acid and distilled water. Both wet and dry weight of purified BCNF were determined, in which the appropriate time for drying is differ according to the thickness of BC membrane formed. Drying of BC membrane was carried out in the oven at 40–50 °C (till reaching a constant weight) then kept in a desiccator with silica gel at room temperature (25 ± 3 °C) for further estimation. BC yield and productivity were calculated as follows:

1 $$Yield \, of \, BC \, \% \, = \, \left[ {\left( {BC \, dry \, wt. \, g/l} \right)/ \, \left( {Original \, sugar \, g/l} \right)} \right] \, \times 100$$

2 $$Productivity \, of \, BC\% \, \quad= \, \left[ {\left( {BC \, dry \, wt. \, g/l} \right)/ \, \left( {Production \, time \, in \, days} \right)} \right] \, \times 100$$

### Gamma irradiation of BCNF

BCNF membranes produced in BT medium were dried, labeled and exposed to different doses of γ-radiation (5.0–25.0 kGy), Indian source (^60^Co-γ) at National Center for Radiation Research and technology (NCRRT), Cairo, Egypt (Dose rate: 0.757 kGy/h).

### Characterization of BCNF

#### Scanning electron microscopy (SEM)

The morphological characters of the bacteria and yeast cells were captured during the growth and synthesis of bacterial cellulose nanofibrils by SEM (ZEISS- EVO 15-UK). On the third and fifth day of fermentation process, a part of the culture media was collected and centrifuged at 4000 rpm for 10 mints and a smear film was prepared on a glass cover slide for examine the cell morphology. While, the smear film of the microbial cells during BCNF synthesis was prepared by taking a loopful from the lower surface of the BC membrane formed on the 5th day of incubation and spread gently to the cover slide. On the other hand, structural investigation of the surface area and cross sections of the dried BCNF films formed on BT medium was also conducted. Samples were coated with gold and an accelerating voltage of 25 kV and magnification of 3000–6000 k were carried out.

#### Fourier transformed infrared spectroscopy (FTIR)

The spectral profile of the dried BCNF was recorded using a BRUKER VERTEX 70 device, in the range 400–4000 cm^−1^. The structural identification of BCNF on SW and BT media were identified compared with that obtained on HS basal medium, while the spectra of BCNF on BT medium were performed before and after irradiation at 5.0–25.0 kGy. All measurements were carried out at room conditions.

#### X-ray diffraction analysis (XRD)

XRD profiles of irradiated and non- irradiated BCNF formed in BT medium were carried out in order to determine its crystalline nature using 6000 SHIMADZU equipped with CuK α radiation source (λ = 0.154 nm) operating at 40 kV and 30 mA (at room temperature). The relative intensity was recorded in the scattering range of (2θ) 5–50° with a step size of 0.05° and a scan speed of 1°min. The effect of different gamma radiation doses on the crystallinity index (CI) of BCNF were calculated from XRD spectrum using Eq. (3) on the basis of the difference between the highest and lowest intensity peaks, where (I_002_) is the intensity of the peak at 2θ = 22° and (I_am_) is the background height between the peaks 2θ = 22° and 2θ = 16°, respectively (Segal et al., 1959).

3 $$CrI \, = \, \left[ {\left( {I_{002} - \, I_{am} } \right)/I_{002} } \right] \, \times \, 100$$

The crystallite size was estimated using Scherrer’s equation:

4 $$CrS \, / \, = \, k \, \lambda \, \left( {\beta \, cos \, \Theta } \right)$$

where, (k) is the dimensionless Scherrer constant = 0.9, (λ) is the X-ray wavelength, (β) is the peak full width at half maximum in radians, and (Ɵ) is the diffraction angle in radians.

All the samples were measured at Central Laboratories (SEM, FTIR and XRD), National Center for Radiation Research and Technology (NCRRT), Egyptian Atomic Energy Authority (EAEA), Egypt.

#### Statistical analysis

All investigations were performed in triplicates and statistical analysis was performed using one-way analysis of variance (ANOVA) by Minitab software (version 17). P value < 0.05 considered as significant (Thomas et al. 2001).

#### Results

### Isolation of bacteria and yeast from Kombucha beverage

**Fig. 1:**
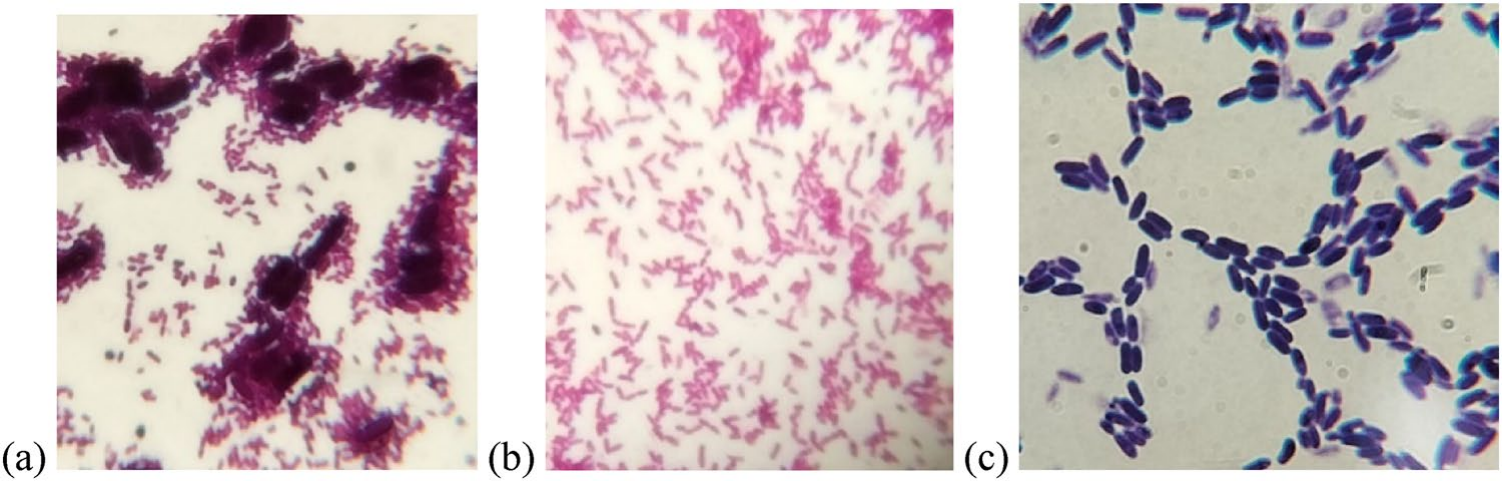
Light microscope of gram stain smear of **a**: symbiotic culture of bacteria and yeast (SCOBY) from Kombucha beverage, **b**: bacterial isolate (KB) and **c**: yeast isolate (KY)

Primary screening of BC production in HS basal medium was evaluated based on the ability to form a white gelatinous membrane on the surface of the medium under static conditions. A gram stain smear from the flasks which showed BC formation was examined by light microscope. As shown in Fig. 1a a symbiotic culture of bacteria and yeast (SCOBY) was illustrated. Separation and purification of bacterial and yeast isolates were carried out on HS and SDA media with antifungal and antibacterial agent, respectively, for further identification. The bacterial isolate was gram negative coccobacilli, while the isolated yeast appeared elongated with budding in some cells (Fig. 1b, c).

**Fig. 2:**
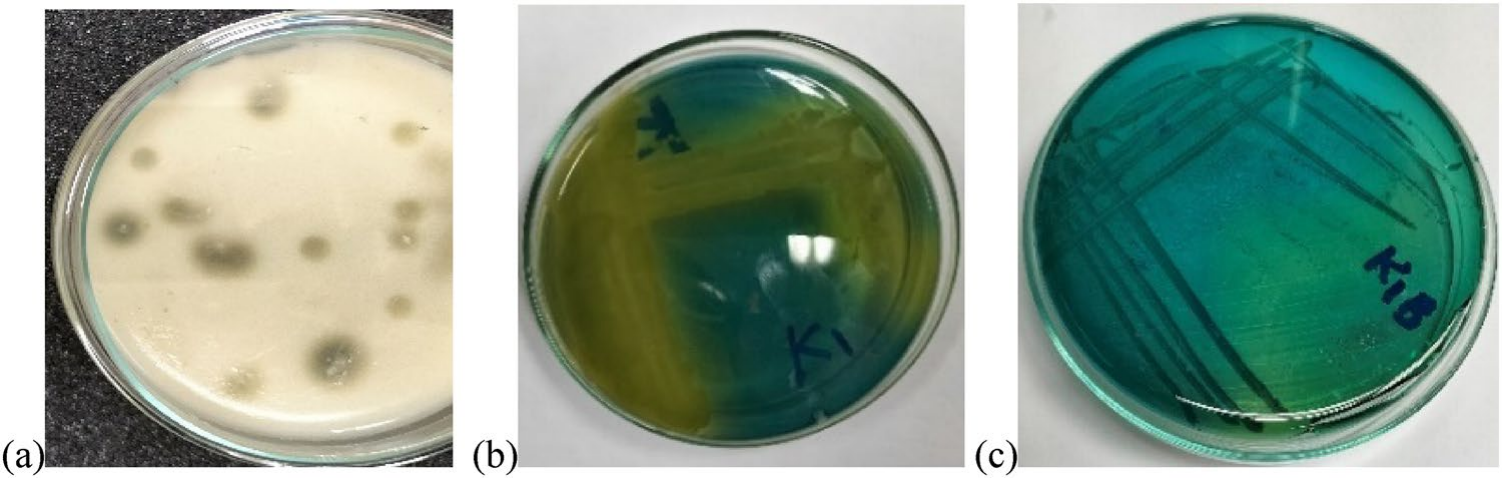
Clear zone surrounding KIB colonies on GYC medium **a**. Color changes of bromocresol green of K1B isolate in Carr medium from green to yellow **b** to green again **c**

The efficiency of bacterial strain isolated from Kombucha beverage (KB) was examined by their ability to produce acid on GYC medium, where the colonies were surrounded by clear zone indicating the hydrolysis of CaCO_3_ as a result of acid production (Fig. 2a). This result was confirmed by the changes in Carr medium color which containing bromocresol purple as indicator. The results showed that the color was changed from green into yellow after 24 h and then into green again (within 48 h) indicating that the isolate is belonging to *Acetobacter* genera (Fig. 2b, c). This result depended on a previous study which reported that the ability of the genera *Acetobacter* to oxidize acetate to CO_2_ and H_2_O was used to distinguish them from *Gluconobacter* members (Carr 1968).

### Identification of bacterial and yeast isolates

**Fig. 3:**
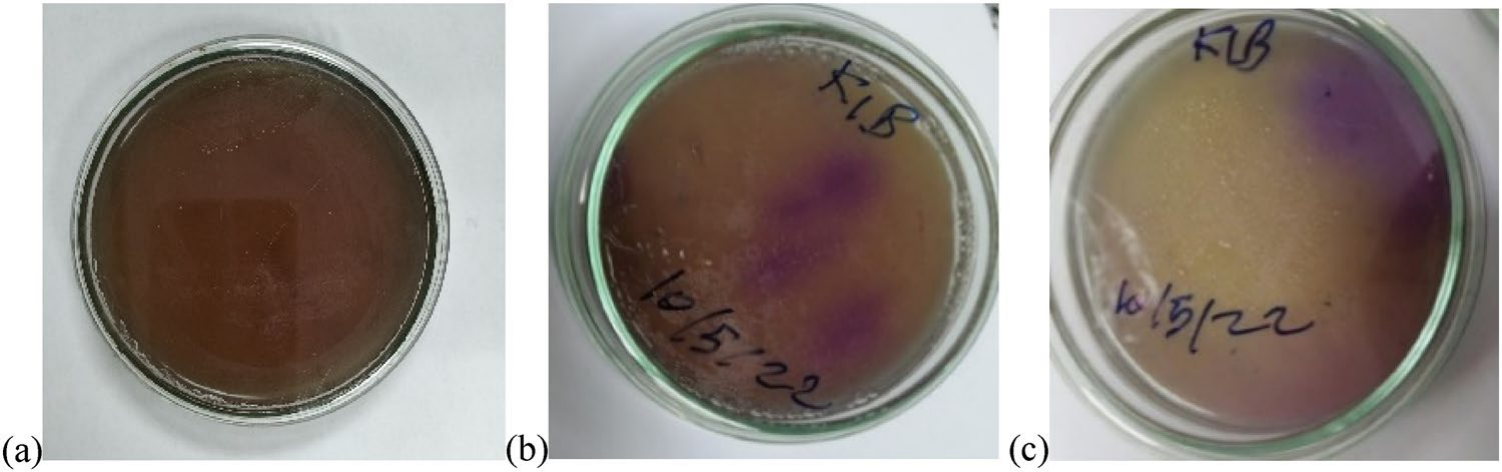
Herellea medium (HM) without inoculation (control) **a**. Pale lavender colonies of *Acinetobacter Spp.* on yellowish background **b**, **c**

The identification of isolated culture by rapid biochemical tests using VITEK2 revealed that the bacterial and yeast isolates are: *Acinetobacter lowffii* and *Candida krusei*, respectively. Herellea was the first medium used for the isolation of *Acinetobacter sp.*, where the colonies of *Acinetobacter* appeared as pale lavender on a yellow background (Jawad et al. 1994), as indicated in the current study (Fig. 3) and confirming VITEK results.

### Production of BC in basal, alternative and modified media

The current study uses BT as an available, simple, cheap and rich components alternative medium for BCNF production compared with three synthetic media (Table 2). The results indicated that there is a significant difference among the tested media, where BT medium gives the best BC production with high dry weight and productivity (3.06 g/l, and 30.6%, respectively). Then, followed by HS and GEM while GACE record the lowest BC dry weight and productivity (0.85 g/l and 8.5%, respectively). It was noticed that the BC membrane formed on the surface of HS medium was appeared as white, loose and thin compared with that formed on BT which was brownish, coherent and thick.

**Table 2:** Yield and productivity of BC production on different media

Media	Sugar conc. (g/l)	Wet wt. (g/l)	Dry wt. (g/l)	Yield (%)	Productivity (%)
HS	20	10.82 ± 0.423^b^	1.8 ± 0.234^b^	9	18
GEM	15	7.78 ± 0.121^c^	1.2 ± 0.142^c^	8	12
GACE	10	5.47 ± 0.474^d^	0.85 ± 0. 068^c^	8.5	8.5
*BT	20	17.63 ± 0.497^a^	3.06 ± 0.205^a^	15.3	30.6

Means with same letters are not significantly different according to Tukey’s test (P < 0.05)

^*^Tea 0.4% (w/v)- 7 days incubation under static conditions at 30 °C

### Different concentrations of Tea and sucrose in BT medium

The effect of different concentrations of tea (0.0–1.0%) and sugar (0.0–10.0%) on BCNF production was illustrated in Table 3. The data revealed that there is no significant difference among the concentration of tea (from 0.2 to 0.8%) and no significant difference was obtained from sugar concentrations (from 4.0 to 10.0%). The concentration of tea and sugar was selected referring to the highest productivity, where 0.2% tea and 6.0% sugar were recorded the optimum results (47.7% and 46.1%, respectively).

**Table 3:** Effect of different concentrations of tea and sugar on BC production (g/L)

Tea conc. (%)	BC dry weight (g/L)	Yield (%)	Productivity (%)	Sugar conc. (%)	BC dry weight (g/l)	Yield (%)	Productivity (%)
0.0	0.787 ± 0.211^b^	1.31	7.87	**0.0**	0.04 ± 0.015^c^	–	0.4
0.2	4.77 ± 0.232^a^	7.95	47.7	**2.0**	3.15 ± 0.275^b^	15.75	31.5
0.4	4.65 ± 0.436^a^	7.75	46.5	**4.0**	4.19 ± 0.513^a^	10.47	41.9
0.6	4.09 ± 0.149^a^	6.81	40.9	**6.0**	4.61 ± 0.055^a^	7.68	46.1
0.8	3.88 ± 0.325^a^	6.46	38.8	**8.0**	4.39 ± 0.435^a^	5.49	43.9
1.0	1.81 ± 0.887^b^	3.01	18.1	**10.0**	4.41 ± 0.474^a^	4.41	44.1

Means with same letters are not significantly different according to Tukey’s test (P < 0.05)

Sucrose 6.0% (w/v)—Tea 0.2% (w/v)- Incubation for 10 days at 30 °C

### Modification of BT alternative medium

A preliminary experiment aiming to optimize the production of BCNF in BT alternative medium was carried out by adding the components of HS medium; other than glucose; in addition to ethanol and acetic acid of GACE medium (Table 4). The data revealed that there is a significant difference among the dry weight of the BCNF produced in BT media according to the additives. It was noticed that the addition of all HS components to BT medium resulted in turbidity, black precipitate and both microbial growth and BCNF production was negatively affected. This result can be attributed to the reaction of HS media components with one or more of tea constituents including; polyphenols, caffeine, adenine, catechins, gallic acids, tannins, amino acids, lipids, chloride, carotenoids, and volatile compounds (Aboulwafa et al. 2019; Dutta and Paul 2019).

**Table 4:** Effect of different supplements in BT on BCNF production (g/L)

Media	Supplements	Dry weight (g/l)	Yield (%)	Productivity (%)	Observation
^a^BT	–	4.77 ± 0.246^d^	7.95	47.7	–
BTHS	HS components	–	–	–	Turbidity and precipitation- inhibit BC formation
BT1	Yeast extract (0.5%)	5.73 ± 0.414^c^	9.55	57.3	Turbidity- increase BC formation
BT2	Peptone (0.5% (	4.33 ± 0.402^d^	7.21	43.3	Turbidity- slight increase BC formation
BT3	Na_2_HPO_4_ (0.27%)	6.84 ± 0.409^b^	11.4	68.4	Change the media to dark brown- enhance BC production
BT4	Citric acid (0.115%)	–	–	–	Change the media to light brown- inhibit BC formation
BT5	Acetic acid (0.01%)	5.05 ± 0.268^cd^	8.41	50.5	No change in color- increase BC production
BT6	Ethanol (1.0%)	7.85 ± 0.225^a^	13.1	78.5	No change in color- enhance and accelerate BC production

Means with same letters are not significantly different according to Tukey’s test (P < 0.05)

^a^*BT* Black tea medium (0.2% tea and 6.0% commercial sugar)

Supplementation of BT medium with ethanol (1.0%), Na_2_HPO_4_ (0.27%), yeast extract (0.5%) and acetic acid (0.01%) individually were resulted in high BCNF production (7.85, 6.84, 5.73 and 5.05 g/l respectively). The results concluded that the addition of ethanol (1.0%) to BT medium not only increase the BCNF production, but also accelerates its synthesis in which the BC membrane started to formed after 2 days of incubation compared with the control (4–5 days).

### Production of BCNF using SCOBY without sterilization

The present experiment was carried out to investigate the probability of BC production without sterilization of BT medium. The flasks showed the formation of BCNF membrane on the surface of the medium after 10 days of incubation, while the flask inoculated by ethanol showed more thick BC membrane. A smear from the flasks was prepared, stained and examined under light microscope to examine the presence of any contaminants. The slides showed no foreign microbial cells among the SCOBY except some residues from the media components.

### Characterization of microbial isolates and BCNF using SEM

**Fig. 4:**
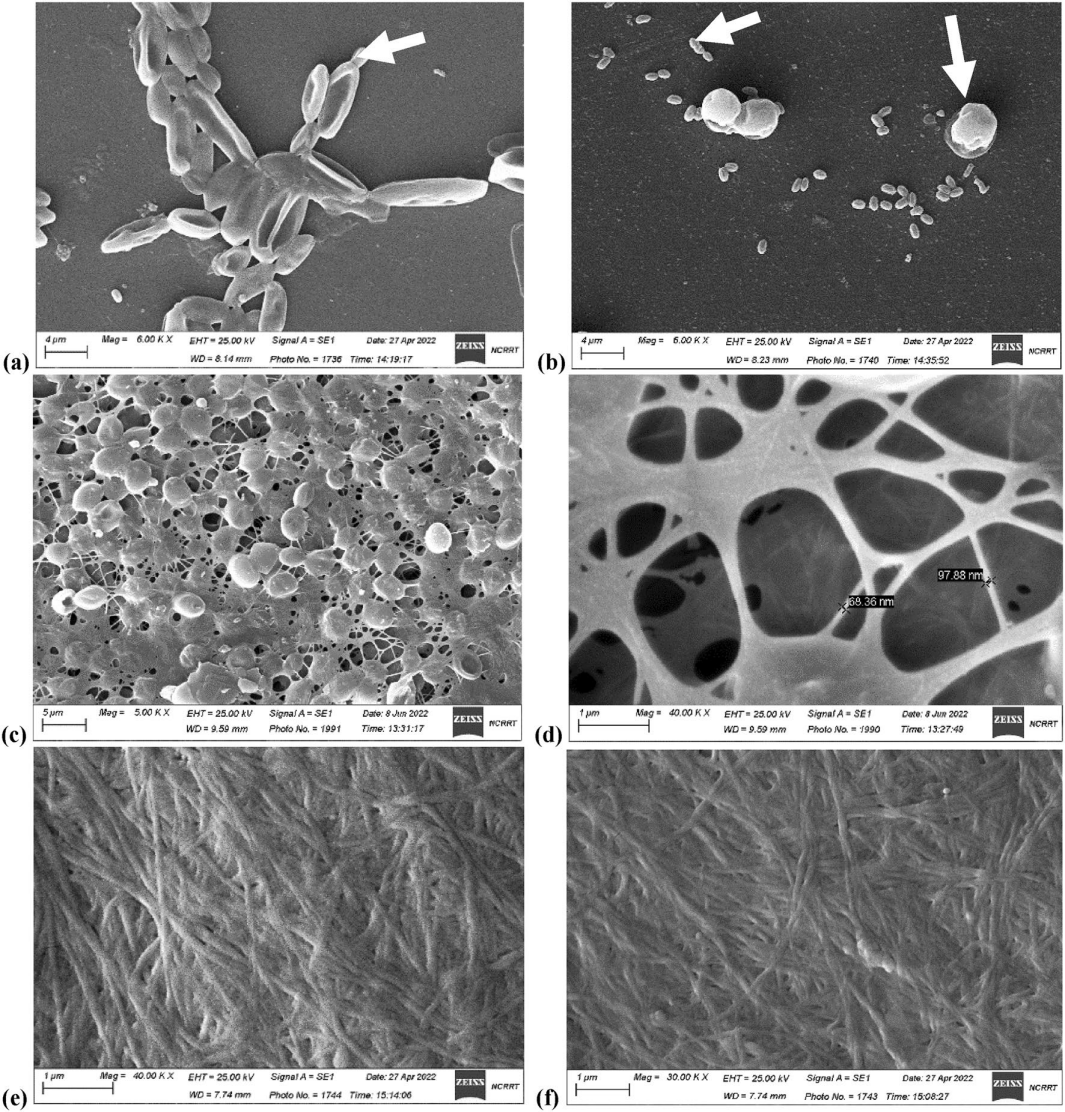
SEM of bacterial and yeast cells in BT medium after 3–5 days **a**, **b**. BCNF surrounding the microbial cells and its nano diameter **c**, **d**. BCNF before and after gamma irradiation at 25 kGy **e**, **f**

The morphological characteristics of bacterial and yeast isolates; *A. lowffii* and *C. krusei (P. kudriavzevii);* were illustrated using SEM. The yeast cells appeared as elongated shape like the cells of *P. kudriavzevii* that mentioned in a previous study (Evy et al. 2013). In Fig. 4a, the arrow pointed to the yeast budding, while the right arrow in Fig. 4b, showed the autolysis of some yeast cells. The left arrow in Fig. 4b pointed out to the bacterial cells which appeared as coccobacilli as described by (Koneman et al. 2006), who reported that *Acinetobacter* often has diploid arrangement.

On the other hand, Fig. 4c and d showed the micrographs of BCNF surrounding the microbial cells during fermentation and the diameter of the fibres was ranged between 68.36 and 97.88 nm. The surface of BCNF was distinguished before and after irradiation at 25 KGy as shown in Fig. 4e and f. The BCNF film without gamma irradiation was appeared as ultra- fine nano-fibrillary structure with irregular fibre arranged in a three-dimensional porous network as mentioned the previous studies (Jung et al 2010; Tsouko et al. 2015). While, the surface of BCNF film became dense and more compacted irradiation at 25 KGy compared to the control film.

### Characterization of BCNF using FTIR

**Fig. 5:**
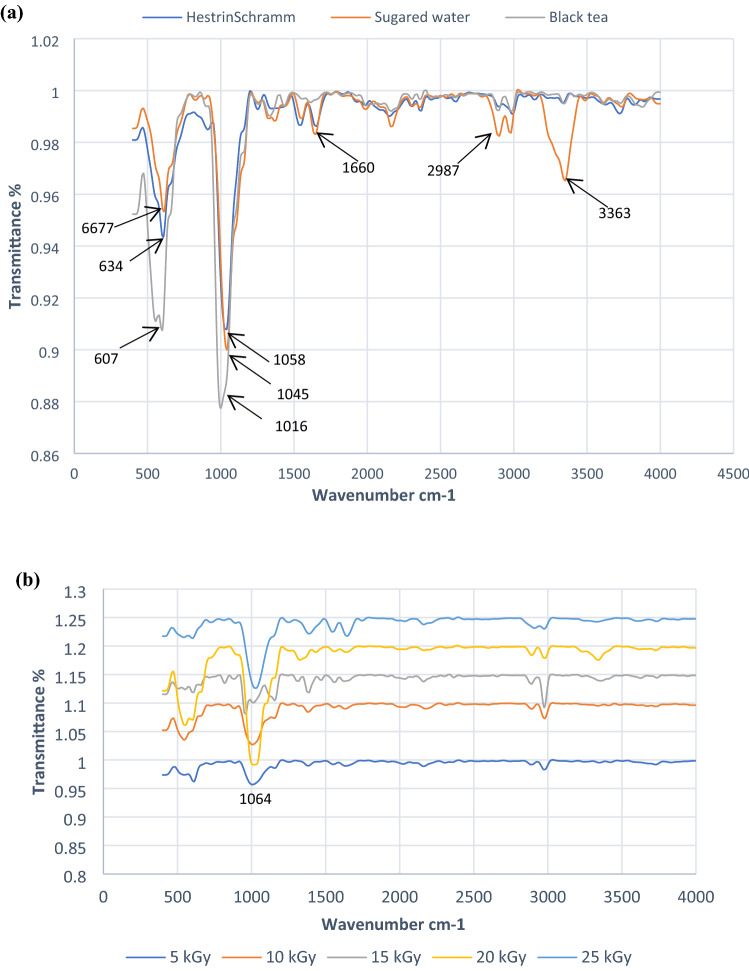
FTIR of BCNF produced by SCOBY on HS, SW and BT media **a**. FTIR of BCNF produced by SCOBY on BT media after exposure to 5, 10, 15, 20 and 25 KGy **b**

Conformational characteristics of BCNF obtained from (HS), (SW) and (BT) media were analysed by FTIR spectrophotometer (Fig. 5). The infrared spectrum displayed an intense absorption peak at the wave numbers 3363 and 2987 cm^−1^ which attributed to stretching valence vibration of free hydroxyl –OH and C–H stretching groups, respectively. While, the peak appeared at 1660 cm^−1^ indicates C = O stretching vibration. The bands in the regions 1016, 1045 and 1055 cm^−1^ corresponded to the elongation of the C–C, C–OH, C–H ring. While C–O–C stretching at the *β* (1–4) glycosidic bond in cellulose and O–H out of phase bending at the wave numbers 607,634 and 667 cm^−1^(Khan et al. 2020). There was an obvious similarity in the spectra of BCNF from the three investigated media. On the other side, FTIR spectra of BCNF after irradiation process at doses 5, 10, 15, 20 and 25 kGy showed the main characteristic absorption bands of BCNF with slight shifts of the peaks, implying the same chemical structure from different culture media.

### Characterization of BCNF using XRD

**Fig. 6:**
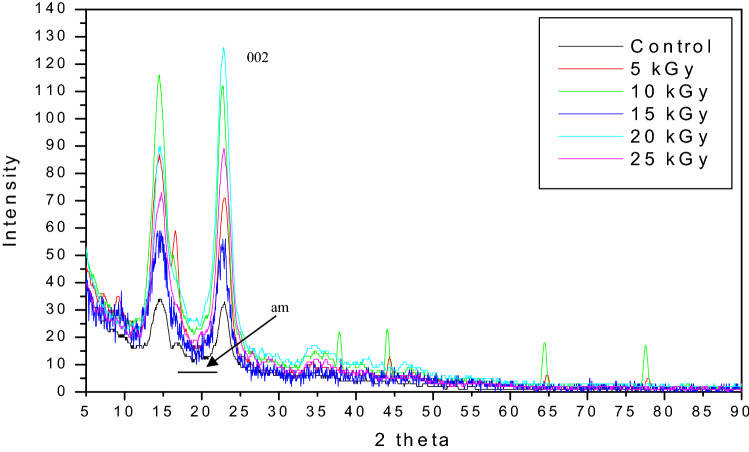
X-ray diffraction (XRD) patterns of non- irradiated and irradiated (5- 25 kGy) BCNF on BT medium

The crystalline structure as well as the change in crystallinity of the BCNF on BT medium was evaluated using XRD analysis. As shown in Fig. 6, the peaks at 2θ angles of 14.54° and 22.84° for the BCNF control confirms the appearance of crystalline type-1 cellulose. A previous study indicated that the peak at around 2θ = 22° is a main crystalline peak (Rosa et al. 2012). After irradiation by 5,10,15,20 and 25 kGy, XRD spectra of BCNF changed evidently and not only the intensity of major peaks increased, but also the specific peaks narrowed and sharped which proved that gamma radiation doses change the crystallinity of BC with different degrees. This result was confirmed by calculating of the crystallinity index (*CrI*) and size (Cs) of the BCNF before and after irradiation as indicated in Table 5.

**Table 5:** The effect of gamma radiation doses on crystallinity index and crystalline size of BCNF in BT medium

Gamma radiation dose (kGy)	I_002_	I_am_	Crystallinity index (*CrI* %)	Difference in *CrI* (%)	Crystalline size (*Cs* in nm)	Difference in *Cs* (%)
0	33.6	11.6	65.5	–	5.96	–
5	71.2	16.6	76.7	17.1	3.96	− 33.5%
10	111.2	22.1	80.1	22.3	5.55	− 6.9%
15	55.2	11.4	79.3	21.1	6.17	3.5%
20	126.3	24.2	80.9	23.5	4.57	− 23.3%
25	88.8	18.3	79.4	21.2	4.43	− 25.7%

The data in table 5 showed a slight fluctuated increase in the *CrI* of BCNF exposed to different doses of gamma radiation in which the percentage of difference was ranged between 17.1 and 23.5%. While, a fluctuated decrease in the crystallinity size (*Cs*) was detected as a result of gamma irradiation doses where the percentage of *Cs* differences was ranged between (− 33.5%) and (− 6.9%). However, the *Cs* recorded a slight increase in size of BCNF after exposure to dose 15 kGy.

## Discussion

Many studies were carried out for biosynthesis of BC from cheap sources and agro-industrial wastes. Most of those studies depend on the production of BC by acetic acid bacteria like; *Acetobacter, Gluconacetobacter* and *Komagataeibacter*. The present study focused on using Kombucha beverage for both isolating the BC producing strains and as a fermentation medium for BC production. A symbiotic culture of bacteria and yeast was isolated from kombucha sample and it were identified as *Acinetobacter lowffii* and *Candida krusei.* This identification was unexpected result because according to the change of color in Carr medium, from green into yellow and into green again, it was supposed that the bacterial isolate was belonging to *Acetobacter genera*. Limited information has been mentioned in the review regarding the use of both *Acinetobacter lowffii* and *Candida krusei* in BC production (Adebayo-Tayo et al. 2017; Teoh et al. 2004).

*Acinetobacter* (order: Pseudomonadales), is a genus of gram-negative non-fermentative, short-rods/coccobacilli, oxidase-negative, catalase-positive and strictly aerobic bacteria (Jaejoon and Woojun 2015). Most of *Acinetobacter sp.* is unable to utilize glucose as a carbon source, while many species are able to partially oxidize several sugars (Oecd 2010). A study hypothesized that a synergistic relationship may develop between *Acinetobacter sp.* and yeast in order to facilitate glucose utilization (Smith et al. 2004).

On the other hand, it was found that the two names *Candida krusei* and *Pichia kudriavzevii* are synonymous with 99.6% identity in DNA sequence for both strains (Kurtzman et al.^1980^; Alexander et al. 2018). *P. kudriavzevii* is widely distributed in nature and approved by the American Food and Drug Administration as “safe” (Bourdichon et al. 2012). It has important applications in biotechnology and food industries (Xiao et al. 2014; De Vuyst et al. 2016; Li et al. 2014; Chelliah et al. 2016) and recently reported for the first time as BC producer (Nurshafiqah et al. 2022).

As the culture medium represents the highest value of the total cost in BC production, therefore using a low-cost alternative culture medium is important in order to increase the yield of BC and expand its commercial production and application area as well. Tea is considered as the second most consumed beverage all over the world after water in addition to its popular use in kombucha beverages. Black tea contains bioactive compounds including; catechins (3.95–38.69 mg/g), flavonoids (6.36–62.10 mg/g), phenolics compounds (11.33–101.29 mg/g) and free amino acids (13.38 mg/g) (D´ebora et al. 2021). These components helped in adjustment the pH of the fermentation medium, enhanced the growth of SCOBY and at the same time inhibit the spoilage with contaminants. In the present study, BT medium proved its capability for BCNF production more than the other synthetic media.

Carbon source is essential for microbial metabolism and growth. In the present study, sucrose represents the main carbon source in BT medium which can be used as substrate for energy and BC production. A previous study found that (9%) sucrose concentration resulted in (66.7%) BC yield and by increasing the sucrose concentration from (11%) to (25%) a gradual decrease in BC yield occurred as a result of gluconic acid production (Goh et al. 2012). The decreases in pH of the culture resulted from gluconic acid produced as a by-product during the fermentation process may affected the bacterial cells survive and subsequently BC formation (Aswini et al. 2020).

The lower BC production resulted from increasing tea concentration may be attributed to the acidic environment or the enzymes released by microbial symbiotic culture which led to the degradation of the complex phenolic compounds and increase the total phenolic contents (Jayabalan et al. 2008).

An important observation showed that; the control flasks containing 4.0% sucrose concentration without tea (sugared water; SW media) showed BC membrane formation. This promising result is indicating the significance of carbon source as a main factor in the BC production and reflects the capability of the current symbiotic culture to form BC in a simple medium depending on the fermentation of sucrose as a sole nutrient and energy source. Similar studies concluded that bacteria and yeast in Kombucha are able to convert sugar and tea under aerobic conditions into carbonated beverage composed of acids, amino acids, vitamins (B1, B2, B6, B12 and C), minerals and some hydrolytic enzymes (Jayabalan et al. 2014; Jayme César et al. 2022).

The suggested mechanism that carried out herein is; sucrose was converted into glucose and fructose via invertase enzyme derived from yeast cells (Ghasemi et al. 2014), which catalysis ethanol production through glycolysis pathway. The produced ethanol utilized as alternative energy source for ATP generation stimulates the bacterial growth and saving glucose to induce the BCNF biosynthesis. While, the nitrogen source and other nutritional requirements were obtained from metabolic products like; vitamins, amino acids and minerals that released in the medium during yeast growth. In addition, the hydrolytic enzymes and metabolic by-products resulted from the autolysis of some yeast cells (as shown in Fig. 5b) may be used as nutrients and stimulate the bacterial growth. This hypothesis is in agreement with the previous studies mentioned that autolysis of yeast cells and metabolic products is considered as a source of nutrients and can stimulate or inhibit the growth of other species in a symbiotic culture (Gomes et al. 2018; Leal et al. 2018).

The obtained results proved that the incorporation of ethanol, yeast extract, acetic acid and di-sodium hydrogen phosphate individually into the sugared tea (BT medium) resulted in increasing the BC production. According to the previous findings, the addition of ethanol can affect BC production by different ways: *(1): ethanol concentration* (0.3‒1.5%) was positively affected the BC production, while the concentration above 2% inhibiting its synthesis (Karin and Kain 2006). *(2): utilization as an energy source* for ATP generation, not as a substrate for BC biosynthesis (Takaaki et al. 1998), which saving the carbon source (sucrose) to the BC production. *(3): control the pathway* by addition of 0.5% ethanol prevents the gluconic acid formation and stimulate the BC production through inhibiting of glucose-6 phosphate dehydrogenase enzyme which shifts glucose-6-phosphate into the pentose phosphate pathway (Mohammad et al. 2015). (*4): prevent the conversion of BC producers into non-cellulose producing mutants* by stimulating the BC produced to aggregate in a cluster around the BC producers and protect them under stress conditions (Park et al. 2003). Another interpretation regarding the effect of ethanol on BC production can be suggested as; the ethanol increase the permeability of the bacterial cell membrane which allow the entrance of more glucose molecules into the periplasmic space where the BC firstly synthesized using cellulose synthase enzyme (Gullo et al. 2018).

On the other hand, the addition of yeast extract in BC production medium has positive effect due to its high nitrogen and growth factors contents (Hegde et al. 2013). This may result in the stimulation and regulation of enzymes involved in the fermentation process and at the same time provide the microbial cells by nitrogen needed for their growth and reproduction. However, the effect of acetic acid in BC production was explained by its consumption to provide energy in the fermentation process and improve the BC production (Dai 2012). The positive effect of organic acids, including acetic acid on BC biosynthesis was illustrated as it causes gluconic acid reduction and direct the pathway towards BC synthesis (Wang et al. 2008; Zhong et al. 2013).While, Na_2_HPO_4_ is used as source of sodium and phosphorus elements, in addition to its use as buffering agent that keep the pH constant in the specific range in the medium to neutralize the acids accumulated during fermentation process that may inhibiting the microbial growth and subsequently affecting the BC production.

As Kombucha tea is worldwide known as a home-made beverage that can be fermented under certain conditions using clean containers without sterilization, therefore an experiment was carried out to investigate the probability of BC production without sterilization of BT medium. The obtained result was in agreement with the previous finding that the symbiotic culture used in the fermentation process in addition to the presence of polyphenolic compounds, ethanol and acids on Kombucha tea resulted in lowering pH during the fermentation process, all of these factors inhibit the growth of pathogenic and contaminating microorganism (Jayabalan et al. 2014; Goh et al. 2012; Sreeramulu et al. 2001, 2000). Moreover, kombucha prepared from both black tea and green tea showed an antimicrobial effect against the tested human pathogenic microorganisms, except *Candida krusei* (Battikh et al. 2012).

Although, no much results were found in the literature regarding the SEM morphology of the isolated SCOBY, but generally both *C. krusei* and *Pichia sp.* have been reported as yeast genera in the Kombucha culture (Marsh et al. 2014; Coton et al. 2017) and the elongated shape of yeast cells was identical to the cell shape of *P. kudriavzevii* (Evy et al. 2013). The autolyzed yeast cells appeared in the smear suggested that the yeast cells start to grow first to convert sucrose into glucose, fructose and ethanol preparing the medium for bacterial growth and BC synthesis, then some of the old yeast cells undergoes to autolysis to give the chance to the bacterial cells to grow and synthesize the cellulose fibers (need more studies for interpretation). This explains the observation of BC membrane in sucrose water (SW) control medium, where the bacterial cells use the debris of the yeast cells after autolysis as nitrogen source and growth factors for growth and BCNF production.

The SEM micrographs of BC captured during the fermentation process (Fig. 5c) showed the nano-fibrillar structure extruded and surrounding the microbial cells forming 3D porous interwoven network, like that obtained in previous studies (Jung et al. 2010; Tsouko et al. 2015). While, the dried BC sheet showed some differences after irradiation which represented in compacted and dense appearance (Fig. 5e, f). The effect of gamma irradiation dose 25 kGy on BCNF dry sheet was selected to be identified by SEM as it is the common and effective dose used for sterilization of pharmaceutical products, biomedical tools and preservation of some foods. The observed differences might be related to the increase of crystalline component due to the formation of the intermolecular bonds created during gamma irradiation (Baccaro et al. 2013). Gamma radiation also has the capability to penetrate the materials, so it is widely used in polymer sterilization and characteristics modification (Benyathiar et al. 2021). It can cause crosslinking or degradation of the polymer chain (Rogers 2012), thereby altering some chemical and physical properties of BC (Salari et al. 2021).

The purity structure and crystalline nature of BCNF support its utilization in various technologies and applications including; biomedical devices, food, cosmetic industries, paper and packaging manufactories; where the purity and strength of the material are required. Accordingly, the confirmational analysis of the BCNF produced by SCOBY on BT and SW by FTIR spectra, showed structure similarity and purity equivalent to that obtained on HS medium as investigated in a previous study (Kumar et al. 2021). The two peaks of (–OH) and (C–H) groups resulted from FTIR spectra are significantly important peaks as they reflect the production of pure and crystalline nano-cellulose (Lin and Dufresne 2014). Moreover, Surma-Ślusarska et al. (2008), reported that bacterial cellulose produced by *Acetobacter xylinum* showed a peak at 3,400 cm^−1^, while another study recorded strong absorption peaks at 3,000–3,700 cm − 1 and 2,800–2,970 cm − 1 indicating the O–H stretching vibration and C–H stretching, respectively (Auta et al. 2017).

However, the chemical and physical properties of polymers can alter as a result from exposure to gamma irradiation which cause crosslinking or degradation of the polymer chain and the same may occur in BC (Rogers, 2012; Salari et al. 2021). FTIR spectra of BCNF after irradiation process showed slight shifts of the peaks, implying the same chemical structure from different culture media. The results demonstrated that sterilization with gamma irradiation up to 25 kGy caused no structural changes in the polymer chains which is in agreement with similar studies (Nascimento et al. 2022; Salari et al. 2021).

On the other hand, the crystallinity is one of the most important features of cellulose which affecting its mechanical and physical properties. As a general, the crystallinity of BC ranged between 60 and 80% (Park et al. 2009) and a previous study investigated that the BC formed in HS medium was 75.6% compared with lower crystallinity (44.22%) in alternative cabbage medium (Nascimento et al. 2022). In the current study, *CrI (*express the relative degree of crystallinity) of BCNF formed on BT medium was calculated from the obtained data of XRD spectra as 65.5%. There was a slight fluctuated increase in *CrI* as a result of exposure to gamma radiation doses with different degrees. This is in agreement with a study concluded that gamma irradiation could increase the crystallinity of bacterial cellulose/chitosan nano composite film (Salari et al. 2021). Another study was conducted by Nascimento et al. (2022), who reported that the *CrI* of BC produced in red cabbage and grapes as alternative medium was decreased in a range of 0.33%– 39.18% after exposure to 25 kGy.

It was found that increasing in crystallinity was referred to the formation of new lamellae, while the decrease in crystallinity was attributed to the crosslinking formation (Alariqi et al. 2009). Both high and low doses of gamma radiation cause destruction of the crystalline structure of kefiran biopolymer. However, the high doses resulted in the formation of mono-, di- and oligo- saccharides, while at low doses the broken structure undergo to recrystallization and formation of crystalline structure with bigger dimensions (Shahabi-Ghahfarrokhi et al. 2015).

Previous studies mentioned that the changes in the XRD spectra of samples irradiated by gamma rays are affected by different parameters including; radiation dose, dose rate, surrounding atmospheric conditions, thickness, density and type of polymer (Shahabi-Ghahfarrokhi et al. 2015, Alariqi et al. 2009). We suggested that the wettability, dryness of BC film and the purification method including NaOH concentration, temperature and time may also affect the crystallinity of BC. Moreover, it was noticed that during fermentation process the formation of unsymmetric thickness BCNF membrane where the edges of the membrane formed at first and extended to the centre in which the membrane appeared with thicker edges than centre. As a general the effect of gamma radiation on BCNF physical and chemical properties still need extra investigations.

## Conclusion

Recently, the attention of researches was increased towards the commercial production of bacterial cellulose for its unique properties and applications. The current study included the isolation of the symbiotic culture (*Acinetobacter lowffii* and *Candida krusei* or *Pichia kudriavzevii*) that were rarely mentioned in the literature as BC producers and succeeded in using sugared black tea (BT) as alternative medium for BCNF production. The symbiotic relationship was captured during SEM examination where lysis of some yeast cells was observed indicating that at certain stage of growth some yeast cells undergo to lysis for saving nutrients in the medium, to provide the bacterial cells by the essential nutrients required for growth and paving the way for BCNF biosynthesis. One of the unexpected results in the current study was the ability of the symbiotic culture to grow and synthesize BC on sugar as sole carbon/energy source indicating the importance of carbon source in BC biosynthesis. The enhancement and acceleration of BCNF biosynthesis by addition of ethanol was explained by increasing the permeability of the cell wall membrane which allows the entrance of more sugar molecules into the periplasmic space where the cellulose synthase present and convert sugars into BCNF. Gamma irradiation affected the crystallinity of BC with different degrees referring to the formation of new lamellae, crosslinking or recrystallization and formation of crystalline structure with smaller or bigger dimensions reflected the crystalline size. This recommends more studies to indicate the relationship between different doses of gamma radiation and the BC properties taking in consideration the production, purification and characterization conditions.

## Data Availability

The datasets generated and/or analyzed during the current study are available from the corresponding author on reasonable request.
